# Efficient T Cell Migration and Activation Require L-Plastin

**DOI:** 10.3389/fimmu.2022.916137

**Published:** 2022-06-29

**Authors:** Hemant Joshi, Sharon Celeste Morley

**Affiliations:** ^1^ Division of Infectious Diseases, Department of Medicine, Washington University School of Medicine, St. Louis, MO, United States; ^2^ Division of Immunobiology, Department of Immunology and Pathology, Washington University School of Medicine, St. Louis, MO, United States

**Keywords:** T cells, L-plastin, immune synapse formation, immune cell adhesion and migration, mechanotransduction, LFA-1 (CD11A/CD18; ITGAL/ITGB2), F-actin assembly, cytoskeleton

## Abstract

Rapid re-organization of the actin cytoskeleton supports T-cell trafficking towards immune sites and interaction with antigen presenting cells (APCs). F-actin rearrangement enables T-cell trafficking by stabilizing adhesion to vascular endothelial cells and promoting transendothelial migration. T-cell/APC immune synapse (IS) maturation also relies upon f-actin-anchored LFA-1:ICAM-1 ligation. Therefore, efficient T-cell responses require tight regulation of f-actin dynamics. In this review, we summarize how the actin-bundling protein L-plastin (LPL) regulates T-cell activation and migration. LPL enhances f-actin polymerization and also directly binds to the β2 chain of the integrin LFA-1 to support intercellular adhesion and IS formation in human and murine T cells. LPL- deficient T cells migrate slowly in response to chemo-attractants such as CXCL12, CCL19, and poorly polarize towards ICAM-1. Loss of LPL impairs thymic egress and intranodal motility. LPL is also required for T-cell IS maturation with APCs, and therefore for efficient cytokine production and proliferation. LPL^-/-^ mice are less susceptible to T-cell mediated pathologies, such as allograft rejection and experimental autoimmune encephalomyelitis (EAE). LPL activity is regulated by its N-terminal “headpiece”, which contains serine and threonine phosphorylation and calcium- and calmodulin-binding sites. LPL phosphorylation is required for lamellipodia formation during adhesion and migration, and also for LFA-1 clustering during IS formation. However, the precise molecular interactions by which LPL supports T-cell functional responses remain unclear. Future studies elucidating LPL-mediated regulation of T-cell migration and/or activation may illuminate pathways for therapeutic targeting in T-cell-mediated diseases.

## Introduction

Activated T cells drive adaptive immune responses. Effective T cell responses require rapid migration towards immune sites, followed by engagement with antigen presenting cells (APCs) (1, 2). By supporting migration and adhesion, cytoskeletal rearrangements regulate the quality and magnitude of the T cell response. The cytoskeleton comprises filamentous proteins including microtubules, actin filaments, and intermediate filaments (3). Receptor engagement, internal organization of the cytoplasm, and stabilization against applied forces trigger cytoskeleton rearrangement (4). For instance, chemokine receptor engagement promotes T-cell motility, shear flow activates T cell adhesion, and T cell receptor (TCR) engagement to peptide-major histocompatibility complex (pMHCs) on APCs triggers immune synapse (IS) formation (5–7).

Actin filaments are the most dynamic cytoskeletal proteins that respond to and direct T-cell biological processes. Actin filaments (f-actin) are formed by the rapid assembly of globular-actin (g-actin) monomers. Bundling, or cross-linking filaments, further remodels f-actin (8, 9). F-actin polymerization is ATP-dependent; ATP binding to monomeric g-actin enhances its affinity for f-actin association (polymerization), while hydrolysis of bound ATP to ADP triggers dissociation (depolymerization). An array of actin-binding proteins regulate actin turnover through g-actin sequestration, nucleation, ATP hydrolysis, nucleotide exchange, f-actin severing, capping, and bundling (10, 11). This review focus on how efficient T cell activation and migration rely on the f-actin bundling protein, leukocyte plastin (L-plastin; LPL).

**Figure 1:**
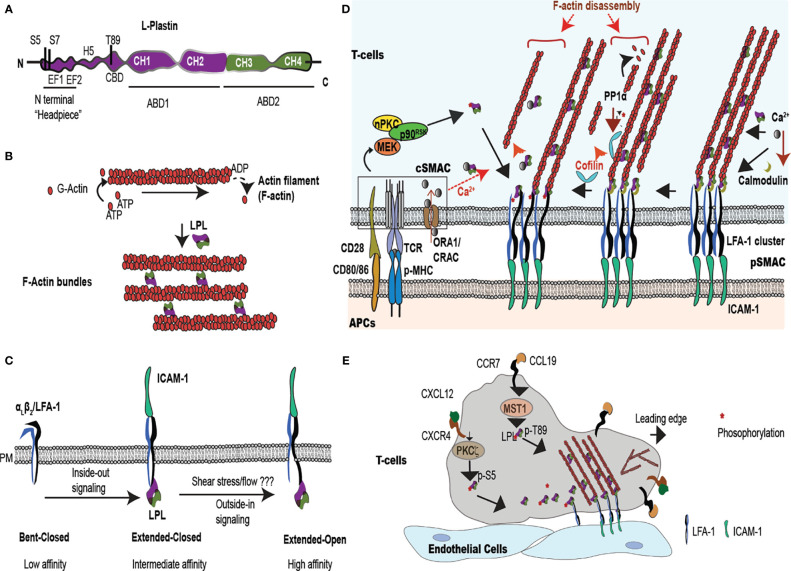
LPL is an actin-bundling protein that supports T cell synapse maturation and migration. **(A)** LPL comprises a regulatory ‘N-terminal headpiece’ and two actin binding domains (ABDs). The regulatory headpiece includes three known phosphorylation sites (Ser-5, Ser-7, Thr89), two calcium binding EF domains, a regulatory H5 “switch helix,” and a calmodulin binding domain (CBD). **(B)** LPL cross-links two actin filaments to generates f-actin bundles. **(C)** LPL interacts with the cytoplasmic tail of the β_2_ subunit of the integrin LFA-1. In T cells, inside-out signaling induces a conformational change from low to intermediate affinity, whereas shear flow or ICAM-1 engagement triggers the high-affinity, extended-open conformation. **(D)** Multiple regulatory pathways converge upon LPL in the pSMAC, where LFA-1 mediates tight approximation to ICAM-expressing APCs. The IS, the interface at the site of T-cell:APC contact site, generates a cSMAC, pSMAC and a dSMAC (dSMAC not shown). In the IS, TCR ligation to peptide-loaded MHC (pMHC) and costimulatory CD28:CD80/86 interactions occur in the central super-molecular activation complex (cSMAC), which activates a kinase cascade [nPKC-MEK- ribosomal protein S6 kinase p90 (p90^RSK^)]. This kinase cascade results in Ser-5 phosphorylation of LPL. LPL phosphorylation accelerates LFA-1 clustering in the peripheral SMAC (pSMAC). Phosphorylated LPL is largely associated with high affinity LFA-1, and may stabilize the IS. LPL can also bind to LFA-1 independently of phosphorylation. LPL-induced LFA-1 clusters further accumulate at IS by cofilin-mediated f-actin remodeling. Cofilin is activated by PP1α phosphatase. Simultaneously, LPL also recruits f-actin bundles to connect the IS to actin cytoskeleton. Ca^2+^ influx at cSMAC also regulates LPL. Ca^2+^ binding reduces the LPL bundling efficiency, perhaps allowing f-actin clearing at IS. However, lower Ca^2+^ in the pSMAC permits calmodulin binding to LPL, stabilizing actin bundles and maintainting LFA-1 clustering. **(E)** Intravascular T cells traffic to peripheral immunological sites *via* the binding of the integrin LFA-1 to the adhesion molecule, ICAM-1, expressed on vascular endothelial cells. In response to chemoattractants, such as CXCL12 and CCL19, T cells polarize the respective chemokine receptors CXCR4 and CCR7 to the leading edge and activate kinases such Mst1 and PKCζ. Mst1, PKCζ, and other kinases phosphorylate the regulatory headpiece of LPL. Phosphorylated LPL initiates LFA-1 clustering, sustaining ICAM-1 binding. Branched f-actin forms in the lamellipod, while the ‘uropod’ propels cells in a forward direction. The symbol "*" indicates the phosphate group during phosphorylation/dephosphorylation reaction.

LPL, also known as lcp1 or plastin-2, bundles f-actin (8, 12, 13). LPL belongs to the plastin family of actin-binding proteins, comprising three isotypes: LPL, T-plastin and I –plastin. Each isoform exhibits distinct tissue expression. Only LPL is expressed in immune cells. Consistent with its expression in hematopoietic cells, LPL specifically binds to β-actin, and does not interact with skeletal muscle α-actin or smooth muscle γ-actin (8). Physiologic LPL expression is restricted to replicating hematopoietic cells, but LPL is also ectopically overexpressed in multiple transformed cancer cells (14). LPL is a 66 kDa protein, consisting of an N-terminal regulatory “headpiece” and 2 actin-binding domains (ABDs); ABD1 and ABD2 (**Figure 1A**). LPL is distinguished from I- and T-plastin by its N-terminal regulatory sequence, as it is the only isoform containing two serine phosphorylation sites at Ser5 and Ser7. Each ABD contains tandem calponin homology (CH) domains (11, 15). How ABD1 and ABD2 of LPL coordinate to engage two actin filaments has not been fully described, because full-length LPL crystallization is unavailable. However, analysis of the available ABD2 structure along with available T-plastin and the *Arabidopsis thalinana* fimbrin (LPL orthologue) protein structures (16) suggests that binding of LPL-ABD2 to f-actin induces “closure” of the actin monomeric ATP binding site. The “closed” conformation inhibits hydrolysis to ADP, reducing depolymerization and stabilizing the actin filament (17). To stabilize bundles, LPL may adopt a twisted conformation, supporting the binding of ABD2 to one filament and activating ABD1 to bind another actin filament, generating cross-linked f-actin (17). Thus, LPL binding stabilizes polymerized actin, as well as altering the twist and tilt of each filament, while LPL bundling arranges filaments into parallel arrays (**Figure 1B**) (16, 18). LPL actin-binding is separable from its function in actin-bundling.

The actin-binding and actin-bundling activities are regulated by the N-terminal headpiece of LPL. The headpiece harbors at least three phosphorylation sites (Ser-5, Ser-7 and Thr-89), two calcium-binding EF-hand loops, and a consensus sequence for calmodulin binding (19–21). Under some conditions, phosphorylation of Ser-5 and Ser-7 enhances actin-bundling activity, and localizes LPL to sites of actin assembly (13, 22, 23). For example, IL-2 stimulation, CD3/CD28 or CD3/CD2 receptor engagement, and CXCL12 treatment all induce Ser-5 phosphorylation (24, 25). However, recent biochemical evidence suggests that single Ser-5 phosphorylation is irrelevant to LPL bundling activity (26), but enhances binding to f-actin structures in non-immune cells and under *in vitro* conditions (13). In T cells, LPL-mediated actin-bundling activity was first noted in a calcium-regulated manner (8). Calcium chelation by the high-affinity EF hands reduces the actin-bundling, but not the actin-binding, activity of LPL (9) (26). Jurkat T cells show increased actin-bundling activity in low intracellular Ca^2+^ concentration of 10^-7^ M, while incubation in higher concentrations, such as 10^-6^ M Ca^2+^, destabilized f-actin bundles (8). Immune cells maintain 10^-8^-10^-7^ M Ca^2+^ in resting phase while elevating to 10^-6^ upon activation, inducing f-actin rearrangement (e.g. intracellular Ca^2+^ is estimated to 50 nM in resting cells and > 1 mM in activated T cells) (27, 28). The physiological range of Ca^2+^ found in T cells correlates with the concentrations of Ca^2+^ that modulate LPL bundling activity. The spatiotemporal regulation of Ca^2+^ flux and LPL activity at the T cell immunological synapse (IS) is discussed in detail later.

In addition to bundling actin, LPL also directly binds to the cytoplasmic tail of the β1 or β2 subunits of integrins. The regulation of integrin affinity and/or avidity by LPL is poorly understood. In brief, integrins are transmembrane heterodimers that bind extracellular matrix proteins or receptors expressed on other cells and mediate cellular adhesion. The dominant integrin mediating T cell adhesion, LFA-1 (α_L_β_2;_ CD11a/CD18), exists in three affinity states: a low-affinity, “bent-closed” state, an intermediate affinity “extended-closed” state, and the high-affinity “extended-open” state (**Figure 1C**) (29). Conversion from the low- to intermediate-affinity state is induced by inside-out signaling; limited evidence suggests that LPL may be dispensable for this conversion (30, 31). However, studies in neutrophils indicated that binding of the β2 cytoplasmic tail by the isolated N-terminal peptide sequence of LPL converted the integrin to a high-affinity state (**Figure 1C**) (32). LPL was also required for integrin-mediated signaling to the oxidative burst in PMNs, though dispensable for integrin-mediated cell spreading (33), indicating that LPL participates in integrin-mediated signaling to downstream events beyond actin remodeling. LPL directly binds the β2 subunit of the integrin LFA-1 to connect with the actin cytoskeleton during migration and activation (34). Finally, LPL was shown to preferentially bind the “clasped” (or closed) cytoplasmic tail of CR3 (Mac-1; α_m_β_2_; CD11b/CD18). Phosphomimetics of Ser5/Ser7 dual phosphorylation reduced the binding of LPL to β_2_, and binding of LPL to β2 reduced CR3-mediated adhesion in the cell line RAW (35). The participation of LPL in T-cell activation and motility therefore extends beyond actin-bundling and into integrin signaling. LPL enables the actin cytoskeletal rearrangements and integrin-mediated adhesion that are core drivers of T-cell migration and T-cell adhesion to APCs and/or target cells. We review the increased knowledge about LPL in T-cell activation and motility, the heart of most adaptive immune responses.

## Requirement for LPL in T-Cell Activation

T cells mount adaptive immune responses by activating other immune cells through cytokine production and/or direct contact, and by direct killing of target cells. TCR engagement by APCs initiates T-cell activation through interaction at a specialized intercellular contact site, the IS. The IS is stabilized by cytoskeleton machinery *via* lamellipodia formation that enhances the contact area between APCs and T cells, increasing TCR signaling (5, 6). The T cell-APC contact site comprises three supramolecular activation clusters (SMAC) that organize signaling proteins into the central, peripheral, and distal SMACs (cSMAC, pSMAC, and dSMAC) (36). The four different actin formations that characterize the IS are extensively reviewed in (36). Notably, the f-actin network is “cleared,” or hypodense, in the cSMAC, where the TCR/CD3 signaling complexes accumulate (37). The pSMAC forms a ring around the centrally cleared area, and is marked by LFA-1 accumulation and generation of actomyosin arcs. These actomysoin arcs are force generating, enabling close approximation of the T cell to its target APC (**Figure 1D**). Larger molecules, such as CD43, localize to the outer dSMAC, where f-actin is organized in a branching network (36, 38, 39). Throughout the pSMAC and dSMAC are small actin foci, areas of integrin engagement that resemble macrophage podosomes. IS formation and T cell activation are absolutely dependent upon active actin rearrangements that create these varied actin structures (13, 40, 41).

Efficient IS formation also requires LPL. Murine T cells isolated from genetically-deficient LPL mice (LPL^-/-^ mice) exhibited impaired IS formation (42), with reduced T-cell:APC contact area. Decreased T-cell activation in LPL^-/-^ mice correlated with enhanced tolerance to T cell-mediated diseases such as skin allograft rejection and experimental autoimmune encephalomyelitis (EAE) (42). Furthermore, LPL supported T_fh_ cell-B cell interactions and was required for germinal center formation and subsequent T-dependent antibody production (43). In human T cells, LPL directly binds the β2 subunit (CD18) of LFA-1 to link the actin cytoskeleton to the IS (34, 44). Reduction of LPL (<10% normal expression through siRNA knockdown), impaired LFA-1 concentration to the pSMAC and generated smaller T-cell:APC contact zone, confirming a crucial need for LPL in IS formation (34).

The requirement for LPL Ser-5 phosphorylation during T-cell activation is unclear. CD3/CD2-mediated Ser-5 phosphorylation was first reported in 1994 (45). Subsequent extensive work using knock-down of LPL in human T cells, followed by re-expression of phosphomimetic or non-phosphorylatable LPL, has suggested a significant regulatory role for Ser-5. In human T cells, Ser-5 phosphorylation enabled translocation of the activation markers CD69 and CD25 to the cell surface after TCR/CD3, CD2 or CD28 engagement, although LPL localized to the IS independently of Ser-5 phosphorylation (24). In the IS, LPL maximizes accumulation of LFA-1 at pSMAC by directly interacting with LFA-1 (34). LPL localization to the pSMAC was stabilized by calmodulin binding and by ABD-mediated actin-bundling activity. LPL bound to LFA-1 equivalently in naive or CD3/CD28-activated T cells, independently of the EF-hand loops and Ser-5 phosphorylation (34). However, Ser-5 phosphorylation of LPL (LFA-1 bound) increased upon CD3/CD28 stimulation and at the IS generated after superantigen [*S. aureus* enterotoxin B (SEB)]-mediated cross-linking of Raji B cells and T cells (31).

Mechanistically, in CD3/CD28-costimulated T cells, activation of the nPKC-MEK- ribosomal protein S6 kinase p90 (p90^RSK^) pathway phosphorylated LPL (Ser-5) to initiate LFA-1 clustering at the IS (31). Association of phosphorylated LPL with high affinity LFA-1 may sustain IS formation (31). Further, LPL-induced LFA-1 clustering was enhanced by another actin-binding protein, cofilin. Cofilin is an f-actin severing protein that promotes f-actin remodeling by generating new filament ends for addition or removal of g-actin monomers (46). Cofilin binds the ADP-bound actin monomer within an existing filament, increasing the likelihood of f-actin depolymerization, accelerating f-actin turn-over, untangling filaments, and facilitating re-arrangement (47). Cofilin enhances LFA-1 accumulation at the IS by accelerating f-actin remodeling (31). Cofilin is activated by PP1α-mediated dephosphorylation, while LPL is dephosphorylated by the serine/threonine phosphatase PP2A (31). LFA-1 clustering and accumulation at the IS enhances the interaction of LFA-1 with its ligand ICAM-1 (expressed by APCs), and amplifies T-cell activation (**Figure 1D**). Pharmacological inhibition of nPKC (Gö6983), MEK (U0126), or p90^RSK^ (BI-D1870) reduced LPL phosphorylation and cofilin activation, which correlated with IS destabilization, diminished T cell-APC contact, and impaired T-cell activation (31). Similarly, blockade of LPL using nanobodies directed against the EF-hands or the ABDs impaired the binding of LPL to LFA-1, and subsequently inhibited IS formation and downstream T-cell proliferation (48). IS stabilization by LPL : LFA-1 binding is targeted by bacterial infections (*e.g. Bordetella pertussis* and *Bacillus anthracis*), in which cAMP production at the IS suppressed T-cell activation (49). Treatment with the glucocorticoid dexamethasone inhibited LPL Ser-5 phosphorylation after CD3/CD2 and CD3/CD28-mediated T cell activation, and dexamethasone also impaired IS formation (50). Thus, a major function of LPL during T cell activation is maintenance of the IS. LPL-mediated IS stabilization is further suggested by the observation that enhanced phosphorylation of LPL amplified LFA-1 clustering and inhibited serial killing by cytotoxic T cells (CTL). Amplified LFA-1 clustering prevented detachment from target cells, thereby prolonging IS maintenance, and thus reduced interactions with new target cells. The pro-oxidative drug WF-10 increased LPL phosphorylation, rigidified the cytolytic IS, and suppressed CTL activity (51). Thus, LPL phosphorylation activates naïve/effector T cells, while interrupting cytolytic activity, making it an important intrinsic modulator during critical immune reactions (44, 51).

Intriguingly, a murine model in which the Ser5 of LPL was converted to the non-phosphorylatable alanine, and thus ablated the Ser5 phosphorylation site, did not exhibit obvious defects in TCR-mediated activation (52). TCR-mediated activation was assessed by proliferation and upregulation of CD25 and CD69. Possible explanations for a lack of effect on T cell activation by ablation of Ser5 phosphorylation include the differences in experimental systems. These explanations will be discussed in detail later.

## Requirement for LPL in T-Cell Recruitment (Migration and Adhesion)

T cells constantly migrate to scan APCs for foreign antigens, to activate immune responses, and to directly kill target cells. This rapid migration (average velocity of 8-15 μm/min) depends on the actin cytoskeleton (27). During chemotaxis, chemokine engagement induces lamellipodia formation and chemokine receptor concentration at the leading edges of cells. Contractile elements at the rear edges, called “uropods,” provide propulsive forces (53). LPL actin-bundling activity is essential for efficient T lymphocyte motility (34, 54, 55).

LPL was first identified as a critical component of T cell chemotaxis during CCL20-induced transwell migration (55). A more detailed analysis of the role of LPL in T cell migration was explored using LPL^-/-^ mice (54). LPL^-/-^ mice showed higher thymic retention of CD4^+^ (3-fold increase) and CD8^+^ (2-fold increase) single positive thymocytes, which resulted from impaired thymic egress. LPL-deficient mature T cells also exhibited impaired intranodal velocity and motility. During CCL19-mediated transwell migration, LPL-deficient T cells showed reduced polarization of CCR7 to the leading edge and reduced uropod formation (54). LPL phosphorylation at Thr-89 by Mst1 kinase (Ste20 kinase/STK4) supported T cell lamellipodia formation and thus T cell migration (21). Mst1 kinase had been previously shown to enable T-cell lamellipodia formation, polarization and migration (13, 56). Identification of a Mst1 consensus sequence in the regulatory headpiece of LPL provided a downstream molecular target by which Mst1 regulated T cell motility. Notably, expression of LPL-T89A, which ablated the Mst1 phosphorylation site (Thr-89), but not Ser-5, failed to restore CCL19-induced CCR7 polarization and transwell migration in LPL-deficient T cells, whereas expression of wild-type LPL did restore the chemokine-induced events. Furthermore, reconstitution of T cells in LPL^-/-^ mice with lentivirally-expressed LPL-T89A showed greater accumulations of CD4^+^ and CD8^+^ single positive thymocytes than did LPL^-/-^ mice, consistent with a requirement for Thr-89 phosphorylation during thymic egress (21).

Investigation in human T cells further confirmed a crucial role for LPL in migration (25). Reduction of LPL by 75-95% by siRNA knock-down caused impaired polarization of CXCR4 receptors in CXCL12 (SDF-1α)-stimulated T cells (25). In human T cells (CD3^+^), CXCL12 activated atypical PKC-ζ to phosphorylate Ser-5 of LPL, triggering lamellipodial localization during polarization (25). However, T cell migration was not impaired in S5A mice, suggesting that Ser-5 phosphorylation of LPL correlates with, but may not be required for, T-cell migration (52). Chemokine signals induce a conformational change in LFA-1, through inside-out signaling, which converts LFA-1 to a moderate-affinity state. Moderate-affinity LFA-1 promotes cell adhesion to vascular endothelial cells *via* ICAM-1 binding (**Figure 1E**). LFA-1:ICAM-1 ligation reorganizes f-actin to initiate T-cell polarization, crawling and migration (57, 58). When LPL expression is reduced, unstimulated and effector (CD3/CD28 stimulated) T cells migrate more slowly and with higher migratory persistence on surfaces coated with immobilized CXCL12 and ICAM-1 (25). Thus, LPL-supported f-actin cytoskeletal polarization is essential for efficient T-cell migration (59).

## Discussion

**Figure 2:**
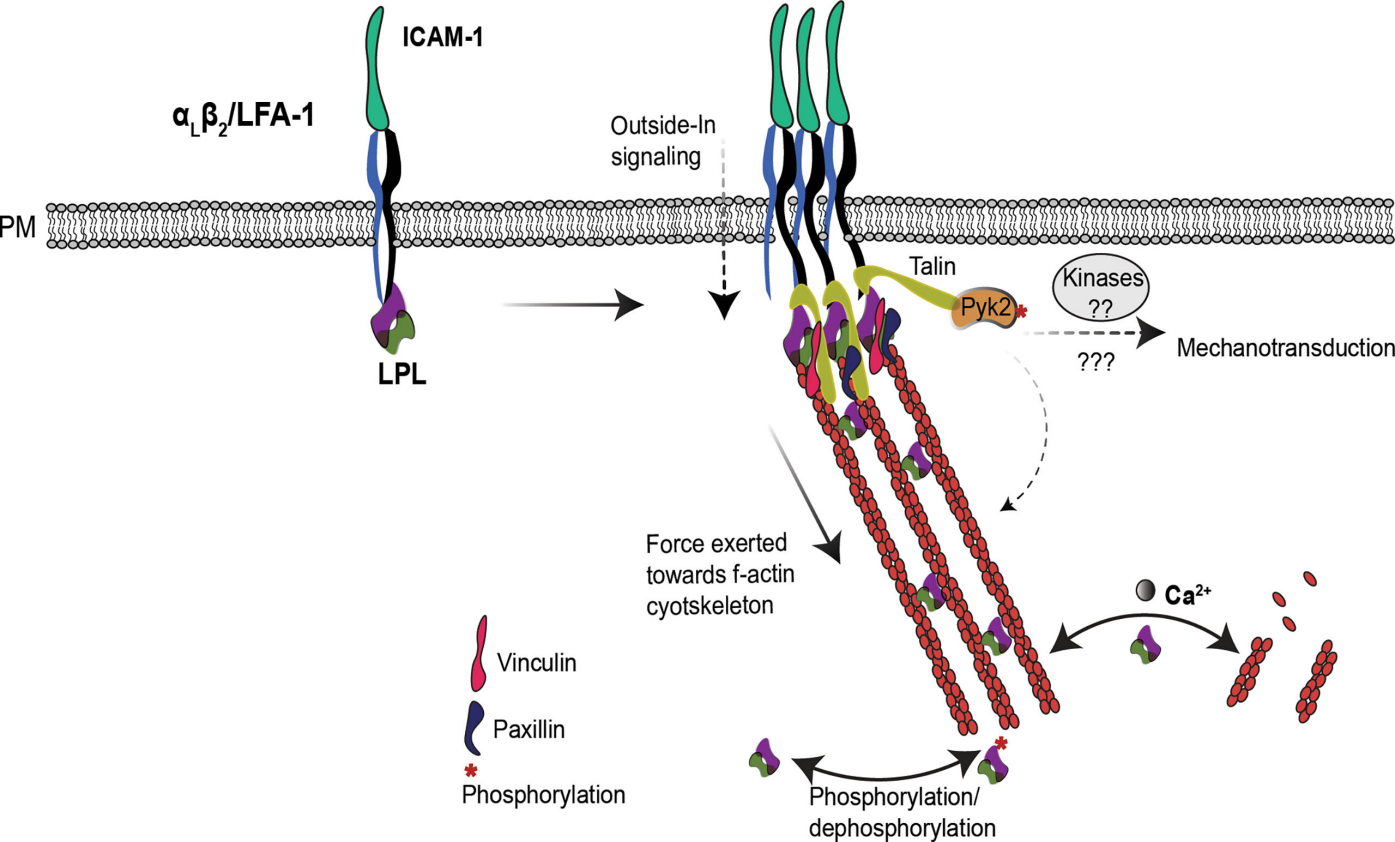
LPL may regulate T cell integrins induced mechanotransduction. In T cells, LPL binds to LFA-1. Under certain conditions, e.g. shear flow or ICAM-1 engagement, LFA-1 shifts to the extended-open (high-affinity) conformation. LPL binding to LFA-1 recruits the mechanosensing protein Talin, and also connects it to the f-actin cytoskeleton. Talin recruits additional cytoskeleton-associated proteins, such as vinculin, paxillin and the tyrosine kinase Pyk2. Collectively, this cascade generates mechanotransduction through Pyk2 and other kinases, and triggers f-actin remodeling required for efficient T cell activation and migration. The separable activities of LPL–LFA-1 binding, f-actin binding, and f-actin-bundling—are coordinately regulated to support mechanotransduction. How the multiple regulatory sites on LPL (phosphorylation, Ca^2+^, and calmodulin-binding) converge to modulate the varied activities of LPL are yet to be illuminated.

A comprehensive model that reconciles apparently disparate observations regarding the regulation of LPL during T-cell migration and activation remains elusive. With multiple regulatory sites, and separable functions of actin-binding, actin-bundling, and integrin-association, too much remains unknown to formulate a complete description of the mechanism(s) by which LPL enables migration and IS formation. To provide a framework for future exploration, we suggest that the primary role of LPL is to stabilize multimolecular complexes that arise at sites of integrin-mediated adhesion, and thereby enhance or promote mechanotransduction at those sites. Mechanotransduction is the translation of external mechanical forces exerted upon the cell into intracellular biochemical signaling (60). For example, IS formation is strengthened by mechanosensation generated by interactions with mature APCs that actively increase cortical cytoskeletal stiffness, thereby boosting T-cell activation (61). Similarly, enhanced adhesion of T cells to stiffer culture surfaces (elastic modulus 25 kilopascal (kPa) to 100 kPa) triggers higher IL-2 production upon CD3 activation (62). Finally, T cells migrate faster on stiffer substrates, demonstrating that mechanotransduction also regulates T cell motility (63). A proposal that LPL supports formation of integrin-mediated adhesion sites and mechanotransduction through these sites provides a single mechanism common to both migration and IS formation (64), and explains how LPL deficiency could disrupt both these processes (**Figure 2**). As discussed below, this framework draws on studies of LPL in hematopoietic cell types including macrophages, B cells, and neutrophils, in addition to T cells.

LPL has been shown to regulate mechanotransduction in macrophages (65). WT macrophages incubated upon softer substrates produced more IL-1β in response to NLRP3 inflammasome activation. However, LPL-deficient macrophages produced the same, reduced amount of IL-1 β following NLRP3 activation, and were unresponsive to varying substrate stiffnesses (65). Mechanotransudction in LPL-deficient macrophages was disrupted due to the mislocalization of the kinase Pyk2. In macrophages, β2 integrin activation phosphorylates Pyk2 to induce podosome signaling (66). Macrophage podosomes are multimolecular signaling complexes generated by integrin-mediated adhesion and anchored by f-actin. Podosomes support macrophage adhesion, signaling and motility. Podosome stabilization requires LPL; thus, LPL also regulates macrophage motility (67). The f-actin foci described as one of the four actin structures present in the T-cell IS have been likened to macrophage podosomes (36). LPL is also required for CXCL12-induced migration and Pyk2 activation (Tyr-402 phosphorylation) in B cells (68). In T cells, Pyk2 regulates LFA-1:ICAM-1 signaling. Pyk2^-/-^ T cells weakly adhere to ICAM-1 and migrate slowly upon CXCL12 stimulation (69), and thus are similar in phenotype to LPL-deficient T cells. While not yet experimentally determined in T cells, it is reasonable to propose that LPL serves to link sites of integrin-mediated adhesion to downstream Pyk2 activation and signaling in all hemaopoietic cells. However, the molecular mechanism by which LPL and Pyk2 interact is unclear, and further investigation in understanding of synergistic functioning of LPL and Pyk2 *via* f-actin cytoskeleton will help in understanding their role in T cell function and mechanotransduction.

In T cells, LPL participates in the “inside-out” cascade for LFA-1 clustering to establish adhesion and IS formation (34). LFA-1 engagement also initiates an “outside -in” signal *via* linking to f-actin (60) to fine-tune T-cell migration, differentiation, and effector functions (70, 71). LPL helps recruit a mechanosensing protein, Talin, to LFA-1 clusters, connecting f-actin to the IS (34). Talin further recruits various cytoskeleton proteins, such as vinculin and paxillin and f-actin, by direct binding, and propagates mechanical signals (72, 73). Since the β_2_-binding site also lies in ABDs (30), it has not yet been shown if a single molecule of LPL can bind both β2 integrin and f-actin, or if binding of one precludes binding of the other. Direct cross-linking of f-actin to the cytoplasmic tail of integrins by LPL would be one possible mechanism by which LPL could sustain integrin-mediated adhesion sites.

During T cell migration, LFA-1 binds to ICAM-1 on endothelial cells to enable transmigration (74). F-actin cytoskeleton retrograde flow aligns ICAM-1 bound integrins at the leading edge to reinforce cellular adhesions (75). While LPL appears to regulate the binding of integrins, such as LFA-1 and VLA-4, to their respective ligands, ICAM-1 and VCAM-1, during chemotaxis on immobilized CXCL12, LPL is dispensable under shear flow conditions (25). This differential requirement for LPL during static chemotaxis and under shear flow could be due to differential f-actin reorganization and/or recruitment of additional actin binding partners, such as Cofilin (31). LFA-1 changes from an inactive, clasped conformation to an active, open conformation to bind ICAM-1 (58, 76, 77). Perhaps the binding of LPL to the intracellular β2 integrin is conformation specific, and varies with T-cell migration under stasis versus shear flow. A recent study in human myeloid (PBL-985) cells showed preferential binding of LPL to the clasped αMβ2 (CD11β/CD18, also called CR3 or Mac-1) conformation to maintain the inactive state under flow (35). By analogy, LPL may likewise stabilize the inactive LFA-1 conformation when T cells are under flow, while under static conditions LPL promotes LFA-1 clustering to initiate adherence. However, conformation-specific binding of LPL to LFA-1 (CD11α/CD18) in T cells is unresolved and future studies will address this important open question.

Further studies are required to determine how signals from the multiple regulatory sites on the LPL N-terminal headpiece are integrated to modify the integrin-binding, actin-binding, and actin-bundling activities of LPL during T-cell activation and migration. Current studies have analyzed single regulatory sites without clearly defining how modulation at other sites impact LPL functioning. For instance, LPL Ser-5 phosphorylation is essential for activation and IS formation in human T cells, while Thr-89 phosphorylation supports T cell migration in murine cells (21). However, no studies have examined the combined effects of Ser5/Thr89 phosphorylation, or if phosphorylation at Ser5 prevents Thr89 phosphorylation. Furthermore, no studies have examined the effect of Ca^2+^ binding on phosphorylated vs non-phosphorylated LPL (at any site). Thr-89 phosphorylation is not yet studied in human T cells, and may not function similarly as it does in mice. Regulation of LPL could vary between human and murine T cells during activation and migration because of different (and as yet undefined) pathways that phosphorylate Ser-5 and Thr-89, or because of different inter-molecular interactions. In human T cells, overexpression of non-phosphorylatable Ser-5 (S5A) LPL abrogated CD3-induced surface translocation of activation markers CD25 and CD69 (24), which was not observed in LPL-S5A reconstituted LPL deficient murine T cells (42). Similarly, our study in LPL-S5A mice showed no defect in CD25 and CD69 upregulation in CD3/CD28 activated T cells (52). However, the exact molecular cascade for LPL-dependent CD69 and CD25 upregulation is not known. Possibly, a dominant negative effect of the S5A mutant in overexpressing human T cells generated a different phenotype than endogenous expression of S5A-LPL-only expressing murine T- cells. Further detailed molecular studies are required to resolve the disparities of LPL regulation in human and mouse T cells.

In addition to phosphorylation, LPL activity is regulated by calcium influx in T cells. The LPL N-terminal headpiece possesses two EF-hand calcium-binding sites and a binding site for the calcium-binding protein calmodulin (15, 78). Calcium binding (increased intracellular calcium concentration above 10^-7^ M) at EF-hands induces a conformational change that inhibits LPL’s bundling activity (8, 9). Recent NMR solution structures of the LPL EF-hand domains revealed a calcium sensor ‘switch-helix’ motif (H5) in-between EF-hand motifs and ABD1 (**Figure 1A**) (79). In the absence of Ca^2+^, the H5 motif remains flexible and unstructured, which stabilizes the orientation of LPL-ABDs when bound to f-actin to promote efficient f-actin bundling. Whereas, when Ca^2+^ increases, the H5 motif changes conformation to a more rigid α-helix, which induces the release of the H5 motif from f-actin-ABD pocket and permits binding of H5 to EF hands. This helix switch in the H5 motif generates a conformation unfavorable to binding of LPL-ABDs to f-actin, thus reducing f-actin bundling (79). The coordinate spatial and temporal dynamics of Ca^2+^ flux and LPL activity during IS formation have not been fully defined. The Ca^2+^ channels ORA1 and CRAC are localized to the cSMAC, suggesting that Ca^2+^ flux is tightly spatially regulated, with increased flux in the cSMAC. Perhaps the higher levels of Ca^2+^ in the cSMAC inhibits LPL bundling activity, contributing to the decreased f-actin polymerization and f-actin density (actin “clearing”) in the cSMAC (37). Lower levels of Ca^2+^ in the pSMAC may correlate with increased LPL-mediated actin-bundling and formation of actomyosin arcs. Calmodulin binding to LPL occurs in the absence of calcium. Calmodulin binding stabilizes LPL binding to LFA-1 clusters in the pSMAC to enhance T-cell/APC contact at IS (34) (**Figure 1D**). Additional studies are required to determine the spatiotemporal regulation of LPL phosphorylation, Ca^2+^ binding, and calmodulin binding during IS formation and maintenance.

The requirement for LPL in T-cell activation reveals new intrinsic regulators for therapeutic targeting to ameliorate T-cell-driven pathologies. For instance, reducing intracellular levels of LPL by genetic knock-down (48) or nanobody-mediated LPL inactivation (48) impair T cell activation and IS formation. Similarly, pharmacological inhibition of LPL directly using dexamethasone (50) or LPL kinase nPKC using Gö6983 (31) can be used to inhibit T-cell immune responses. Conversely, enhancing LPL phosphorylation using WF-10 could be employed to suppress T cell mediated cytotoxicity during allograft transplantation (51). In addition to activation, inhibiting cell migration by targeting kinases [e.g. Mst1 (21) and PKCζ (25)] could be explored to maximize T-cell retention in tumors microenvironment. The urgent need for therapies for autoimmune and oncologic processes compels further elucidation of the key T-cell migration and activation pathways dependent upon LPL.

## Author Contributions

HJ and SM both have equally contributed to idea conceptualization and manuscript writing. All authors contributed to the article and approved the submitted version.

## Funding

This work was supported by National Institutes of Health grants R01- AI104732, R21EB030171, R01 AI118719, HL148033, R01AI139540, and R56 AI104732, National Science Foundation grants CBET-1900277 and CMMI1548571, American Lung Association grant ETRA 736343, Washington University in Saint Louis Children’s Discovery Institute grants CDI-CORE-2019-813 and CDI-CORE-2015-505, Foundation for Barnes-Jewish Hospital grants 3770 and 4642, American Association of Immunologists AAI Careers in immunology fellowship. The content of this study is the authors’ sole responsibility and does not necessarily represent official NIH views.

## Conflict of Interest

The authors declare that the research was conducted in the absence of any commercial or financial relationships that could be construed as a potential conflict of interest.

## Publisher’s Note

All claims expressed in this article are solely those of the authors and do not necessarily represent those of their affiliated organizations, or those of the publisher, the editors and the reviewers. Any product that may be evaluated in this article, or claim that may be made by its manufacturer, is not guaranteed or endorsed by the publisher.
